# Bis(β-lactosyl)-[60]fullerene as novel class of glycolipids useful for the detection and the decontamination of biological toxins of the *Ricinus communis* family

**DOI:** 10.3762/bjoc.10.155

**Published:** 2014-07-03

**Authors:** Hirofumi Dohi, Takeru Kanazawa, Akihiro Saito, Keita Sato, Hirotaka Uzawa, Yasuo Seto, Yoshihiro Nishida

**Affiliations:** 1 Department of Nanobiology, Graduate School of Advanced Integration Science, Chiba University, 1-33 Yayoi-cho, Inage-ku, Chiba 263−8522, Japan; 2 Department of Materials and Life Science, Shizuoka Institute of Science and Technology, 2200-2 Toyosawa, Fukuroi, Shizuoka 437-8555, Japan; 3 National Research Institute of Police Science, 6-3-1 Kashiwanoha, Kashiwa, Chiba 277-0882, Japan; 4 Nanosystem Research Institute, National Institute of Advanced Industrial Science and Technology (AIST), 1-1-1 Higashi, Tsukuba, 305-8565, Japan

**Keywords:** fullerene, multivalent glycosystems, oligosaccharides, proteotoxins, ricin

## Abstract

Glycosyl-[60]fullerenes were first used as decontaminants against ricin, a lactose recognition proteotoxin in the *Ricinus communis* family. A fullerene glycoconjugate carrying two lactose units was synthesized by a [3 + 2] cycloaddition reaction between C_60_ and the azide group in 6-azidohexyl β-lactoside per-*O*-acetate. A colloidal aqueous solution with brown color was prepared from deprotected bis(lactosyl)-C_60_ and was found stable for more than 6 months keeping its red color. Upon mixing with an aqueous solution of *Ricinus communis* agglutinin (RCA_120_), the colloidal solution soon caused precipitations, while becoming colorless and transparent. In contrast, a solution of concanavalin A (Con A) caused no apparent change, indicating that the precipitation was caused specifically by carbohydrate–protein interactions. This notable phenomenon was quantified by means of sodium dodecyl sulfate polyacrylamide gel electrophoresis (SDS-PAGE), and the results were discussed in terms of detection and decontamination of the deadly biological toxin in the *Ricinus communis* family.

## Introduction

Carbohydrate-binding proteins (lectins) and proteotoxins, e.g., verotoxins [1–2] and cholera toxins [3], can cause serious damages to human cells. The carbohydrate binding proteins are able to interact with cell-surface glycoconjugates such as glycoproteins and glycolipids to aggregate the cells. Proteotoxins penetrate into the target cells after binding with glycoconjugates and disturb vital cell functions. Ricin, a proteotoxin isolated from the castor bean of the *Ricinus communis* family, is one of the strongest biological toxins and is registered as a scheduled compound in the Chemical Weapon Convention [4]. Ricin consists of a subunit A with ribonuclease activity and a subunit B possessing carbohydrate-binding domains specific to β-lactosyl linkage [5–6]. In the past years, the development of proteotoxin infection inhibitors based on carbohydrate molecules has attracted great interest [7–8]. In particular, multivalent biomaterials carrying more than two carbohydrate ligands have been designed [9–15] and proven to enhance protein–carbohydrate interactions by means of glycocluster effects [16–18].

More recently, our research group has reported on attempts of applying these glycomaterials for both the detection and the decontamination of biological toxins in an assumed polluted scene [19–21]. In the present study, we attempted to apply our *N*-glycosyl-[60]fullerenes [22–25], which were designed as a novel class of glycolipids with notable biological and physical properties. For example, bis(α-D-mannopyranosyl)-[60]fullerene is capable of forming a liposome-like supramolecule in aqueous media and exhibits a strong binding activity to an α-mannose-binding lectin (concanavalin A, Con A) as the result not only of carbohydrate cluster effects but also of a unique spatial arrangement of the bis(mannosyl) linkage on the [60]fullerene surface [25]. In this paper, we describe our first synthesis of bis(β-lactosyl)-[60]fullerene and its potential as a tool for detecting and decontaminating the deadly biological toxin, ricin.

## Results and Discussion

**Figure 1:**
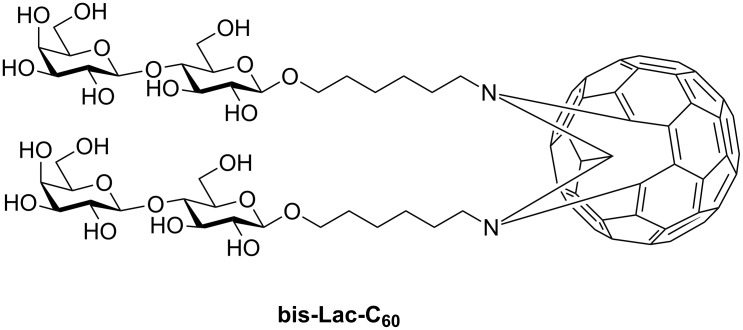
Structure of bis(β-lactosyl)-[60]fullerene (bis-Lac-C_60_).

In our preceding studies [24–25], we have shown that bis(α-mannosyl)-[60]fullerene can be obtained by a coupling reaction between 1-azidoalkyl per-*O*-acetyl-glycoside and C_60_ together with [5,6]- and [6,6]-junction isomers of mono(α-mannosyl)-[60]fullerene. The bis(glycosyl)adduct is more polar than the two monoadducts and can be easily separated by silica gel column chromatography. Taking these preceding results into account, we prepared the bis(β-lactosyl)-[60]fullerene (bis-Lac-C_60_, Figure 1) in the present study.

### Synthesis of bis(per-*O*-acetyl-β-lactosyl)-[60]fullerene **4**

**Scheme 1:**
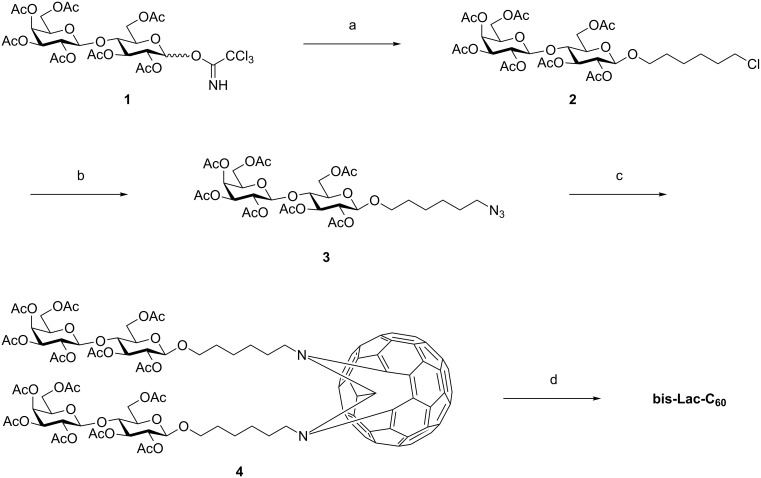
Synthesis of bis-Lac-C_60_. Reagents and conditions: (a) 6-chloro-1-hexanol, TMSOTf, CH_2_Cl_2_, −40 °C, 1 h, 48%; (b) NaN_3_, DMF, 60 °C, 16 h, 93%; (c) C_60_, chlorobenzene, reflux, 7 h, 14%; (d) NaOMe, CH_2_Cl_2_, MeOH, 5 h.

The bis(lactosyl)-fullerene has been prepared from lactosyl trichloroacetimidate **1** [26] following a pathway as shown in Scheme 1 [25]. The coupling reaction between **1** and 6-chloro-1-hexanol was conducted in the presence of trimethylsilyl trifluoromethanesulfonate (TMSOTf) to yield β-lactoside **2**. The nucleophilic substitution of the terminal chloride group in **2** with sodium azide afforded glycosyl azide **3**. The thermal cycloaddition of the azide group to C_60_ was conducted by boiling in chlorobenzene to obtain a mixture of the three main products, which were identified as a mixture of [5,6]- and [6,6]-fused isomers of monoadducts and the targeted bisadduct **4** in TLC analysis. **4** was purified with chromatography on silica gel and identified with NMR and MS spectroscopy as the desired bis(per-*O*-acetyl-β-lactosyl)-[60]fullerene (Experimental and Supporting Information Information file 1).

### Preparation of colloidal suspension of bis(β-lactosyl)-[60]fullerene (bis-Lac-C_60_)

All acetyl groups in **4** were removed with sodium methoxide in a mixture of dichloromethane and methanol. During this process, the reaction mixture deposited aggregates of bis-Lac-C_60_, which were collected by filtration and washed thoroughly with methanol. The aggregates were diluted with dimethyl sulfoxide (DMSO) and dialyzed against distilled water for 2 days to give a colloidal suspension of bis-Lac-C_60_ with a deep brown color. The derived suspension was stable for at least 6 months when stored at 4 °C in the dark. Dynamic light scattering (DLS) analysis indicated that the colloidal suspension might involve spherical particles with two different sizes, smaller particles with a diameter range of 30–50 nm (av 39.6 ± 6.7 nm) and larger particles with a diameter range of 150–170 nm (av 162 ± 29 nm). We observed analogous results for colloidal suspensions of mono- and bis(α-D-mannopyranosyl)-[60]fullerenes in both AFM (atomic force microscopy) and DLS analyses. Probably, the smaller particles are bilayer vesicles that are stable in DMSO and pyridine while they can be destructed in parts by treatment with surfactants such as Triton-X [25]. These nano-sized constructs tend to attract each other to form the larger particles, and this tendency seems to be pronounced in polar aqueous solvents.

### Precipitation assay for the colloidal suspension of bis-Lac-C_60_ with proteins of the *Ricinus communis* family

**Figure 2:**
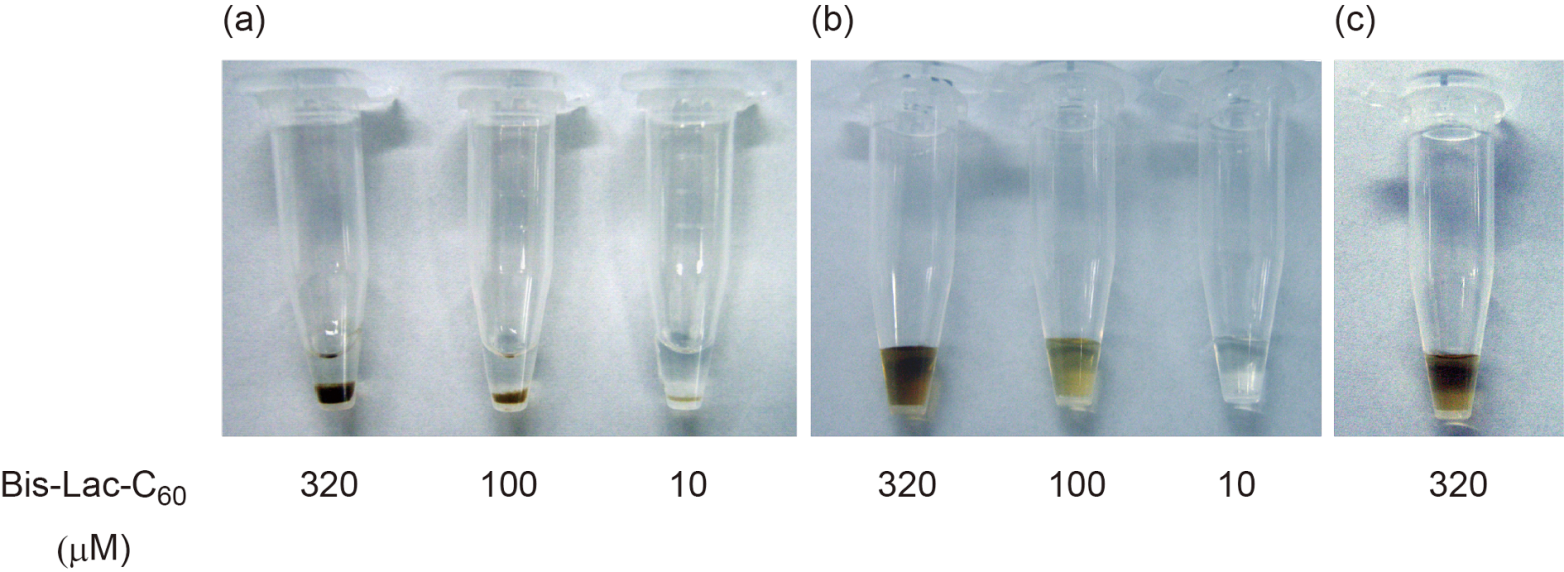
Precipitation assay of bis-Lac-C_60_ colloidal solution. The tubes were allowed to stand for 10 min after the addition of proteins or buffer. (a) 10 μL of 20 μg mL^−1^ RCA_120_ solution; (b) 10 μL of 10 μg mL^−1^ Con A solution; (c) 10 μL of 100 mM PBS buffer.

With the colloidal suspension of bis-Lac-C_60_ in hand, precipitation tests were conducted with *Ricinus communis* agglutinin (RCA_120_) [27], ricin and concanavalin A (Con A) [28]. RCA_120_ is a ricin-like lectin and able to bind β-galactose residues. Con A is an α-mannose-specific lectin. When RCA_120_ (10 μL, 20 μg mL^−1^) was added to this suspension (0.1 mL, 300 μM), the suspension soon gave rise to dark brown precipitate (Figure 2a). The precipitate was collected by centrifugation, washed thoroughly with water, and then applied to SDS-PAGE. A clear band was observed at the region matching with RCA_120_, supporting that this lectin was directly associated with the sedimentation. Also upon the addition of ricin, a similar phenomenon could be observed, even though the precipitation took a prolonged time (ca. 15 min) in comparison with the case of RCA_120_ (ca. 2 min). Apparently, the proteotoxin possesses a lower ability to crosslink the [60]fullerene vesicles into larger sediments, though the reason is unknown. In contrast, no precipitation could be found in the negative control, which were composed of the same PBS buffer solution albeit free from these proteins. In addition, Con A in the same PBS buffer solution could not induce any sedimentation (Figure 2b and Figure 2c). These observations allowed us to expect that the sedimentary phenomenon might arise from species-specific interactions of the *Ricinus communis* proteins with the lactose cluster arrayed on the surface of the [60]fullerene vesicles. In nature, there are a lot of β-lactose-binding proteins. Not only to the *Ricinus communis* proteins but also to many other β-lactose or β-D-galactose-binding proteins, the β-lactose cluster arrayed on the surface of the [60]fullerene vesicles may become an ideal ligand. In this context, the sedimentary reaction is not specific to the proteins from the *Ricinus communis* family. Probably, in an assumed polluted scene, the colloidal suspension of the bis(β-lactosyl)-[60]fullerene will be useful to check the presence of ricin-like proteins.

### Quantitative analysis of ricin protein in the bis-Lac-C_60_ colloidal suspension by means of SDS-PAGE

**Figure 3:**
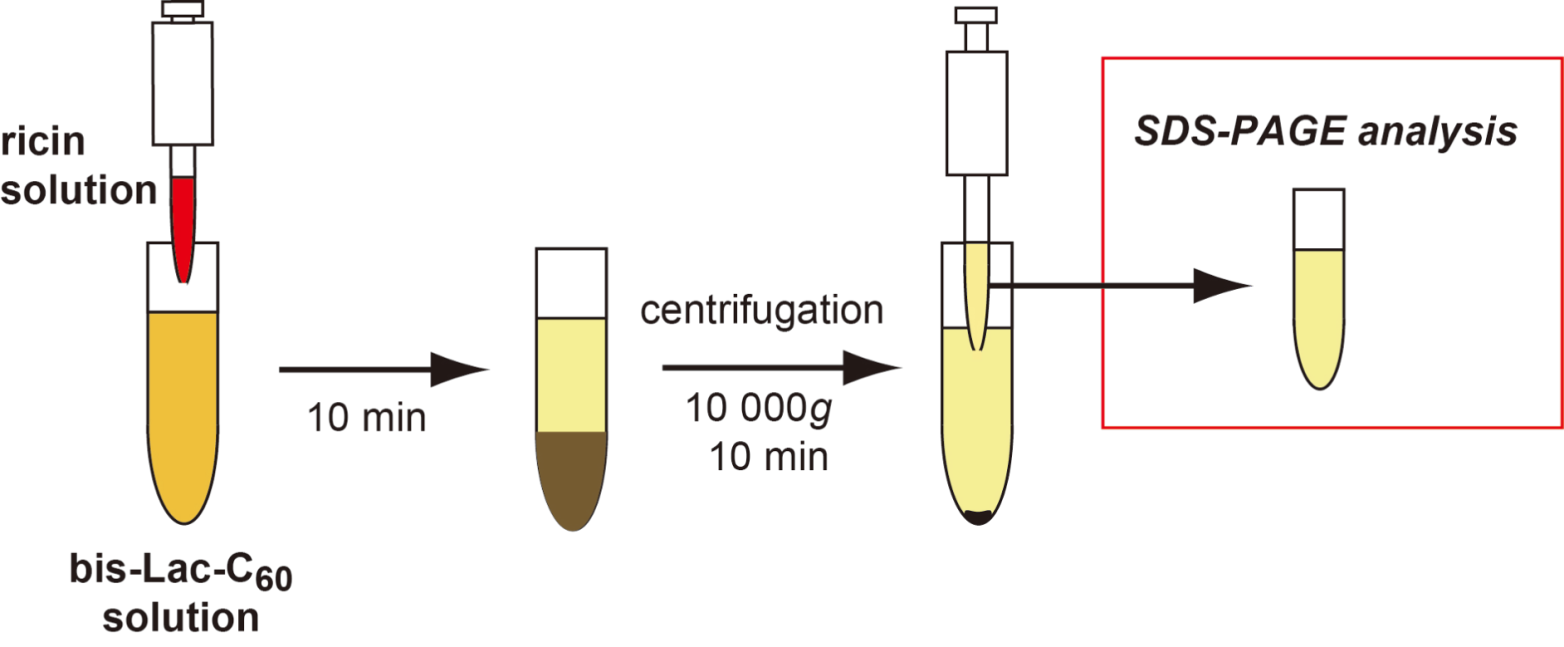
Schematic image for the quantitative analysis of ricin protein in the colloidal suspension of bis-Lac-C_60_.

The above results have suggested that the *Ricinus communis* toxins and probably also other lactose-binding proteins can crosslink the vesicles of bis-Lac-C_60_ and then deposit aggregates at the bottom. If this holds true, the vesicles of bis-Lac-C_60_ can serve as decontaminants to remove ricin and related proteins from dangerous areas contaminated with biological toxins. In this section, we report on the examination of the behavior of ricin protein in the bis-Lac-C_60_ suspension by estimating its distribution (%) in both the supernatant and the aggregate after the sedimentation. The test samples were prepared in a manner as summarized in Figure 3. A ricin solution was added to the suspension of bis-Lac-C_60_ at different concentrations in the range of 1–100 μM. The mixtures were allowed to stand for 10 min and then centrifuged at 10 000*g* for 10 min. The amount of ricin remaining in the aqueous phase was quantified from the intensity of the protein band in SDS-PAGE. The amounts were calibrated with standard samples with known concentrations.

Though Bradford and Lowry methods might be useful for this kind of protein assays, the strong UV–vis absorbance of the C_60_ chromophore interfered with these established methodologies. Therefore, we undertook an alternative way by means of the SDS-PAGE.

The results summarized in Table 1 show that the ricin protein was partitioned into two phases, i.e., solid phase (precipitates) and liquid phase (supernatants), after the sedimentation. Its distribution (%) in the solid phase increased with the concentration of bis-Lac-C_60_. At 100 μM, most of the protein (94%) was deposited at the bottom as aggregates (run 4 in Table 1). These results support our previous suggestion that the sedimentary reaction in the colloidal suspension is based on toxin–lactose interactions and thus is useful for a simple detection of the biological toxin.

**Table 1:** Distribution (%) of ricin protein (0.1 mg mL^−1^) after sedimentation in the colloidal suspension of bis-Lac-C_60_ at different concentrations.

run	bis-Lac-C_60_ (μM)	distribution of ricin (%)	
		precipitate	supernatant

1	1	26	74
2	10	31	69
3	50	74	26
4	100	94	6

### Glyco-nanotechnology for locking the deadly toxin at the bottom

**Figure 4:**
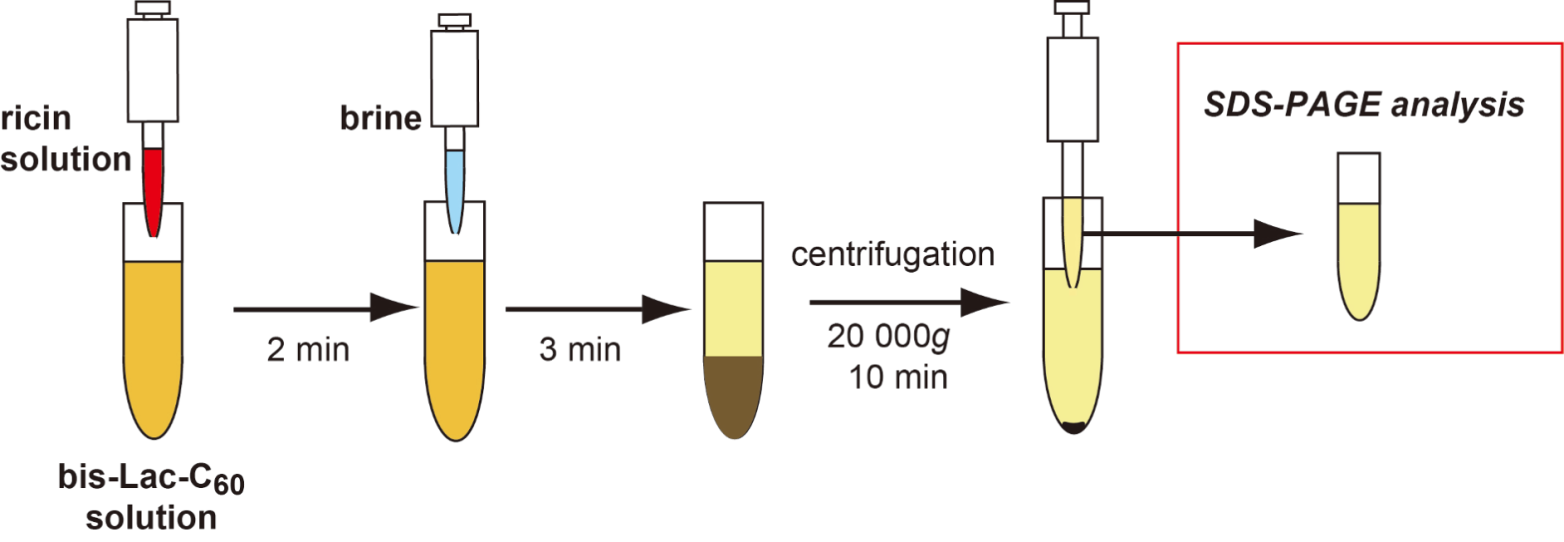
A modified procedure for the rapid detection and the efficient decontamination of ricin and ricin-like proteins.

In an assumed situation of bioterrorism, the total time required for the identification and the decontamination is one of the key factors for minimizing possible damages from contaminated biological toxins. Obviously, a simple and highly effective method is required for this purpose. We have recognized in the above study that the colloidal suspension of bis-Lac-C_60_ can deposit ricin in more than 90% efficiency in a structural form of “protein–lactose aggregates.” This means that the bis(β-lactosyl)-[60]fullerene can provide us with a promising tool to tackle the deadly toxin. At the end of this study, we attempted to establish our protocol for the rapid detection and the efficient decontamination of ricin and ricin-like proteins. The overall protocol examined here is schemed in Figure 4. Though this is similar to that already shown in Figure 3, the total manipulation time was shortened to 20 min and the decontamination efficiency was improved by a brine-induced salting-out effect.

First, an aqueous ricin solution was added to the colloidal suspension of bis-Lac-C_60_. For the first 2 minutes, the suspension gave no apparent sediment. Upon addition of brine, the mixture soon generated precipitates. After standing for another 3 minutes, the mixture was centrifuged and analyzed with SDS-PAGE in the same manner as described previously (see also Experimental). By changing concentrations of both brine and bis-Lac-C_60_ solutions, we optimized the conditions for locking this toxin at the bottom effectively.

The results are summarized in Table 2. At a constant bis-Lac-C_60_ concentration (183 μM or 363 μM), the decontamination efficiency (%) increased with the concentration of brine. The efficiency reached 99% at 500 mM brine concentration and 363 μM bis-Lac-C_60_ (run 6 in Table 2). Consequently, the modified procedure enabled us to decontaminate ricin with >99% efficiency within 20 min.

**Table 2:** Efficiency (%) in the decontamination of ricin.

run	bis-Lac-C_60_ (μM)	brine (mM)	efficiency^a^ (%)

1	183	100	89.1
2	183	200	97.9
3	183	500	98.4
4	363	100	89.3
5	363	200	97.8
6	363	500	99.3

^a^The efficiency (%) was determined from the distribution (%) of ricin partitioned in the aqueous phase after sedimentation and centrifugation.

## Conclusion

A bis(β-lactosyl)-[60]fullerene was synthesized and evaluated as a novel class of glycolipid in the form of a red-colored colloidal suspension in aqueous medium. Its potency was obvious in the precipitation assay by using *Ricinus communis* proteins, which soon precipitated at the bottom while the red-colored suspension changed to colorless transparent solution. The observed phenomenon, which is based on multivalent protein–lactose interactions, prompted us to apply this glycolipid as a tool for the rapid detection and the decontamination of ricin and other biological toxins. By using an SDS-PAGE analysis, we successfully quantified distributions (%) of ricin in the aqueous and the solid phase. With this analytical tool in hands, we have also optimized the reaction conditions and proposed two protocols. The first protocol facilitates the detection, the second protocol allows for both the detection and the decontamination. The latter enabled us to deposit the toxin at the bottom of polluted solutions with efficiency greater than 99%. Obviously, the lactosyl-[60]fullerene provides us with a simple and powerful tool for tackling such dangerous toxins that aggregate our cells and/or penetrate into cells by a common way of protein–carbohydrate interactions.

## Experimental

**Safety consideration:** Ricin is a highly toxic protein and was used with permission from the Minister of Economy, Trade and Industry of Japan. It should be handled using protective clothing in a fume hood, and should be decontaminated with an autoclave apparatus after examination.

**General:** All reactions were conducted under a dry argon atmosphere. All chemicals involved in the bis(lactosyl)-fullerene synthesis were purchased from Wako Pure Chemical Industries Co., Ltd., Tokyo Chemical Industry Co., Ltd. (Japan) and Sigma-Aldrich Co. (USA) and used without further purification. All reactions were monitored by thin-layer chromatography (TLC) on an aluminum sheet silica gel (60 F_254_ Merck, Sigma-Aldrich) by using UV-light detection and ethanolic phosphomolybdic acid or a *p*-anisaldehyde solution and heat as developing reagents. Flash column chromatography was performed on a silica gel (Merck 60 Å, particle size: 0.040–0.063 mm) by using toluene/ethyl acetate, hexane/ethyl acetate, cyclohexane/ethyl acetate, and chloroform/methanol mixtures as eluents. ^1^H NMR (500 MHz), ^13^C NMR (125 MHz), and 2D NMR spectra were recorded with a JNM-LA-500s or JNM-ECA-500 spectrometer (JEOL, Japan). Unless otherwise stated, NMR spectra were recorded at 22 °C in CDCl_3_ with tetramethylsilane (TMS) as an internal standard and a digital resolution of 0.30 Hz. The following abbreviations correspond to spin multiplicities: s = singlet, d = doublet, t = triplet, m = multiplet, br = broad, and bs = broad singlet. FABMS spectra were recorded on a JEOL JMS-AX-500 spectrometer. HRMS–ESI spectra were recorded with a Thermo Scientific Exactive mass spectrometer. FTIR spectra were recorded on a JASCO FTIR-230 spectrometer (Japan) as KBr films. Ricin (2.5 mg mL^−1^) was obtained from Honen Corporation (now J-Oil Mills, Inc., Tokyo, Japan) in a 10 mmol L^−1^ potassium phosphate buffer (pH 7.2) containing 0.8% (w/v) sodium chloride and 0.02% (w/v) potassium chloride.

**6-Chlorohexyl 2,3,6,2’,3’,4’,6’-hepta-*O*-acetyl-β-lactoside (2):** A suspension of glycosyl imidate **1** (4.65 g, 5.96 mmol), 6-chloro-1-hexanol (2.37 mL, 17.9 mmol), and molecular sieves 4 Å (3.00 g) in dichloromethane (30 mL) was stirred for 1 h. After cooling to −40 °C, TMSOTf (53.8 μL, 0.298 mmol) was added to the suspension and the mixture was stirred for another 1 h. After quenching with triethylamine, the reaction mixture was diluted with chloroform and filtered through a Celite pad. The filtrate was washed with brine, the organic layer was dried over MgSO_4_, filtered, and concentrated in vacuo. The residue was chromatographed on silica gel by using cyclohexane/ethyl acetate 3:2 to give glycoside **2** as a white solid (2.16 g, 48%): [α]_D_ −11.4 (*c* 1.0, CHCl_3_); ^1^H NMR (500 MHz, CDCl_3_) δ 5.35 (dd, *J*_3’,4’_ = 3.4 Hz, *J*_4’,5’_ = 0.9 Hz, 1H, H-4’), 5.19 (t, *J*_2,3_ = *J*_3,4_ = 9.2 Hz, 1H, H-3), 5.11 (dd, *J*_1’,2’_ = 7.9 Hz, *J*_2’,3’_ = 10.7 Hz, 1H, H-2’), 4.97 (dd, *J*_2’,3’_ = 10.4 Hz, *J*_3’,4’_ = 3.4 Hz, 1H, H-3’), 4.88 (dd, *J*_1,2_ = 7.9 Hz, *J*_2,3_ = 9.5 Hz, 1H, H-2), 4.49 (d, *J*_1’,2’_ = 7.9 Hz, 1H, H-1’), 4.49 (m, 1H, H-6a), 4.45 (d, *J*_1,2_ = 7.9 Hz, 1H, H-1), 4.15–4.06 (m, 3H, H-6b, H-6’a and H-6’b), 3.89–3.82 (m, 2H, H-5’ and –C*H*_2_–), 3.79 (t, *J*_3,4_ = *J*_4,5_ = 9.7 Hz, 1H, H-4), 3.60 (dq, *J*_4,5_ = 9.8 Hz, *J*_5,6_ = 2.1 and 4.9 Hz, 1H, H-5), 3.53 (t, *J* = 6.7 Hz, 2H, –C*H*_2_–Cl), 3.49–3.44 (dt, *J* = 6.4 and 9.5 Hz, 1H, –OC*H*_2_–), 2.15, 2.12, 2.06, 2.04, and 1.97 (s×5, 15H, –OCOC*H*_3_), 2.05 (s, 6H, –OCOC*H*_3_), 1.79–1.73 (m, 2H, –C*H*_2_–), 1.61–1.54 (m, 2H, –C*H*_2_–), 1.46–1.41 (m, 2H, –C*H*_2_–), 1.37–1.32 (m, 2H, –C*H*_2_–); ^13^C NMR (125 MHz, CDCl_3_) δ 170.35, 170.31, 170.12, 170.04, 169.77, 169.56, 169.06, 101.05, 100.55, 76.29, 72.81, 72.58, 71.70, 70.97, 70.66, 69.85, 69.10, 66.59, 62.00, 60.77, 44.90, 32.44, 29.20, 26.46, 25.08, 20.82, 20.77, 20.66, 20.58, 20.46; HRMS–FAB (*m*/*z*): [M + K − H]^+^ calcd for C_32_H_47_O_18_Cl, 793.2088; found, 793.2040.

**6-Azidohexyl 2,3,6,2’,3’,4’,6’-hepta-*O*-acetyl-β-lactoside (3):** A suspension of compound **2** (2.16 g, 2.86 mmol) and sodium azide (372 mg, 5.72 mmol) in *N*,*N-*dimethylformamide (10 mL) was stirred at 60 °C for 16 h. The reaction mixture was diluted with ethyl acetate and the organic layer was washed with brine twice, dried over MgSO_4_, filtered, and concentrated in vacuo. The residue was chromatographed on silica gel by using toluene/ethyl acetate 1:1 to give azide **3** as a white solid (2.02 g, 93%). [α]_D_ −11.1 (*c* 1.13, CHCl_3_); ^1^H NMR (500 MHz, CDCl_3_) δ 5.31 (dd, *J*_3’,4’_ = 3.4 Hz, *J*_4’,5’_ = 0.9 Hz, 1H, H-4’), 5.16 (t, *J*_2,3_ = *J*_3,4_ = 9.5 Hz, 1H, H-3), 5.08 (dd, *J*_1’,2’_ = 8.0 Hz, *J*_2’,3’_ = 10.6 Hz, 1H, H-2’), 4.92 (dd, *J*_2’,3’_ = 10.3 Hz, *J*_3’,4’_ = 3.4 Hz, 1H, H-3’), 4.85 (dd, *J*_1,2_ = 7.7 Hz, *J*_2,3_ = 9.5 Hz, 1H, H-2), 4.46 (d, *J*_1’,2’_ = 8.0 Hz, 1H, H-1’), 4.46 (m, 1H, H-6a), 4.42 (d, *J*_1,2_ = 7.7 Hz, 1H, H-1), 4.12–4.03 (m, 3H, H-6b, H-6’a and H-6’b), 3.86–3.78 (m, 2H, H-5’ and –C*H*_2_–), 3.76 (t, *J*_3,4_ = *J*_4,5_ = 9.5 Hz, 1H, H-4), 3.56 (dq, *J*_4,5_ = 9.7 Hz, *J*_5,6_ = 2.0 and 5.2 Hz, 1H, H-5), 3.53 (dt, *J* = 6.6 and 9.5 Hz, 2H, –C*H*_2_–N_3_), 3.23 (t, *J* = 6.9 Hz, 1H, –OC*H*_2_–), 2.12, 2.03, 2.00, 1.93, and 2.09 (s×5, 15H, –OCOC*H*_3_), 2.01 (s, 6H, –OCOC*H*_3_), 1.55 (m, 4H, –C*H*_2_–), 1.33 (m, 4H, –C*H*_2_–); ^13^C NMR (125 MHz, CDCl_3_) δ 170.41, 170.21, 170.13, 169.86, 169.64, 169.14, 101.14, 100.64, 76.39, 72.88, 72.66, 71.78, 71.05, 70.73, 69.95, 69.17, 66.67, 62.09, 60.87, 51.39, 29.30, 28.83, 26.44, 25.47, 20.95, 20.89, 20.77, 20.71, 20.58; HRMS–ESI (*m*/*z*): [M + Na]^+^ calcd for C_32_H_47_N_3_O_18_, 784.2752; found, 764.2747.

***per*-*O*-Acetyl bis-Lac-C_60_ 4:** A suspension of compound **3** (588 mg, 0.772 mmol) and C_60_ (278 mg, 0.386 mmol) in chlorobenzene (117 mL) was stirred until complete dissolution of C_60_. The mixture was freeze-deaerated and heated under reflux for 7 h. After cooling to ambient temperature, the mixture was concentrated in vacuo. The residue was purified by silica gel column chromatography by using a toluene:ethyl acetate gradient (1:0→3:1→2:1→1:1) to afford bisadduct **4** as a black solid (243 mg, 14%) and compound **3** (179 mg, 30%): ^1^H NMR (500 MHz, CDCl_3_) δ 5.35 (d, *J* = 3.1 Hz, 1H, H-4’), 5.21 (t, *J*_2,3_ = *J*_3,4_ = 9.3 Hz, 1H, H-3), 5.11 (dd, *J*_1’,2’_ = 7.9 Hz, *J*_2’,3’_ = 10.4 Hz, 1H, H-2’), 4.96 (dd, *J*_2’,3’_ = 10.4 Hz, *J*_3’,4’_ = 3.4 Hz, 1H, H-3’), 4.90 (dd, *J*_1,2_ = 8.3 Hz, *J*_2,3_ = 9.5 Hz, 1H, H-2), 4.50–4.48 (m, 3H, H-1, H-6, and H-1’), 4.16–4.00 (m, 4H, H-6, H-6’×2, and –C*H*_2_– ), 3.91–3.77 (m, 3H, H-4, H-5’, and –C*H*_2_–), 3.63–3.58 (m, 1H, H-5), 3.55–3.46 (m, 1H), 2.15, 2.13, 2.06, 2.05, and 1.97 (s×5, 21H, –OCOC*H*_3_ ), 1.49 (b, 1H, –C*H*_2_–), 0.87 (b, 1H, –C*H*_2_–); ^13^C NMR (125 MHz, CDCl_3_) δ 170.32, 170.31, 170.11, 170.03, 169.77, 169.55, 169.05, 147.59, 146.81, 145.10, 144.97, 144.86, 144.60, 144.54, 144.17, 144.11, 144.09, 143.94, 143.72, 143.53, 143.33, 142.66, 142.00, 141.55, 141.23, 139.54, 139.32, 138.95, 138.79, 137.11, 135.18, 134.56, 132.86, 130.48, 129.01, 128.19, 125.27, 101.07, 100.62, 77.22, 76.31, 72.84, 72.62, 71.73, 70.97, 70.64, 70.02, 69.69, 69.10, 66.57, 62.01, 60.75, 51.60, 29.47, 29.41, 29.38, 27.09, 25.82, 20.92, 20.84, 20.78, 20.64, 20.51; HRMS–ESI (*m*/*z*): [M + Na]^+^ calcd for C_124_H_94_N_2_O_36_, 2209.5484; found, 2209.5467.

**Bis−Lac C_60_:** Sodium methoxide (5 mg, 93 μmol) was added to a solution of **7** (7 mg, 3 μmol) in dichloromethane (2 mL) and methanol (0.5 mL), and the mixture was stirred. The reaction was monitored by TLC and FTIR visualization of the decrease of the peak originating from the carboxyl group. After 5 h of stirring, a black precipitate was collected by filtration and washed with methanol to give bis-Lac-C_60_ as a black solid.

**Preparation of the colloidal suspension of bis-Lac-C_60_:** Bis-Lac-C_60_ (7 mg, 3 μmol) was dissolved in dimethyl sulfoxide (2 mL), and the solution was poured into a dialysis tube (Cellulose Dialyzer Tubing VT351, molecular weight cut-off: 3500, Nacalai Tesque, Inc., Japan) suffused with distilled water (20 mL). After 2 days of dialysis, the solution was subjected to ultrafiltration at 3,000*g* for 15 min by using an Amicon Ultra-15 device (molecular weight cut: off 5000, Millipore, Co., USA). The concentrate was transferred into a measuring flask, and the total volume was adjusted with distilled water to give a bis-Lac-C_60_ dispersion colloidal suspension at the desired concentration.

**Precipitation assay for the colloidal suspension of bis-Lac-C_60_ with proteins of the *Ricinus communis* family:** A solution of RCA_120_ in water (10 μL, 20 μg mL^−1^), Con A in water (10 μL, 10 μg mL^−1^), or PBS buffer (10 μL, 100 mM) was separately added to the 320 μM colloidal suspension of bis-Lac-C_60_ (100 μL) in Eppendorf tubes. The mixtures were vigorously shaken by means of a vortex mixer and allowed to stand for 5 min before careful physical examination.

**SDS-PAGE analysis of the precipitate generated by the addition of RCA_120_ solution to the colloidal suspension of bis-Lac-C_60_:** An RCA_120_ solution (60 μL, 1 mg mL^−1^) in water was added to a bis-Lac-C_60_ colloidal suspension in water (300 μM, 940 μL), and the mixture was vigorously shaken by using a vortex mixer and allowed to stand for 10 min. The mixture was centrifuged at 10,000*g* for 20 min, and the supernatant was removed. Water (1 mL) was added to the residual black pellet, which was vigorously dispersed. The black suspension (10 μL) was mixed with a buffer containing SDS (10 μL), and the mixture was heated to 90 °C for 10 min. An RCA_120_ solution (1 mg mL^−1^) was also denatured by the same procedure. Each solution (10 μL) was applied to the polyacrylamide gel (14%) and electrophoresed for 1 h. The gel was dyed with Coomassie Brilliant Blue (CBB).

**Quantitative analysis of ricin in the colloidal suspension of bis-Lac-C_60_:** A ricin solution (1.67 mg mL^−1^, 60 μL) was added to each bis-Lac-C_60_ colloidal suspension (940 μL), and the mixture was shaken vigorously and allowed to stand for 10 min. After centrifugation of the mixture at 10,000*g* for 10 min, the supernatant (100 μL) was collected and concentrated with a centrifugal vacuum concentrator. Ricin solutions (100 μL) at concentrations of 50, 25, 10, 5, 1, and 0.5 μg mL^−1^ were also prepared to construct the calibration curve and concentrated with a centrifugal vacuum concentrator. All concentrated residues were denatured with SDS (20 μL) at 90 °C for 10 min, and each solution (10 μL) was applied to the polyacrylamide gel (14%). The gel was dyed with Flamingo solution, and band intensities were estimated by using a laser excitation imaging kit. The residual ricin concentration in the bis-Lac-C_60_ colloidal suspension was determined by means of the calibration curve, which shows the ricin intensities at each concentration.

**Estimation of decontamination efficiency by using a salting-out agent:** A ricin solution (2.5 mg mL^−1^, 60 μL) was added to each bis-Lac-C_60_ solution (940 μL), and the mixture was shaken and allowed to stand for 2 min. Brine (100 μL) was added to the mixture, shaken vigorously, and allowed to stand for 3 min. After centrifugation of the mixture at 20,000*g* for 10 min, the supernatant (100 μL) was collected and concentrated with a centrifugal vacuum concentrator. Ricin solutions (1 mL) at concentrations of 50, 25, 10, 5, 1, and 0.5 μg mL^−1^ were prepared and separately mixed with brine (100 μL) as control solutions. These solutions (100 μL) were collected and concentrated with a centrifugal vacuum concentrator, respectively. Subsequent procedures to determine the concentration of ricin were carried out according to the protocol mentioned in the previous section. The decontamination efficiency against ricin (%) was calculated by the formula [ricin concentration of centrifuged supernatant (μM)/concentration of initial ricin solution (μM)] × 100 (%).

## Supporting Information

File 1 Copies of ^1^H and ^13^C NMR spectra for compounds **2**, **3** and **4**.
