# Supplementary material for: Bis(β-lactosyl)-[60]fullerene as novel class of glycolipids useful for the detection and the decontamination of biological toxins of the Ricinus communis family
Source: Beilstein J Org Chem. 2014 Jul 3;10:1504–12. doi: 10.3762/bjoc.10.155 (PMC4142837; doi:10.3762/bjoc.10.155)
Supplement: File 1 — Copies of 1H and 13C NMR spectra for compounds 2, 3 and 4. [file Beilstein_J_Org_Chem-10-1504-s001.pdf]

Supporting Information

for

Bis(β-lactosyl)-[60]fullerene as novel class of glycolipids useful for detection and decontamination of biological toxins of the *Ricinus communis* family

Hirofumi Dohi[1*], Takeru Kanazawa[1], Akihiro Saito[2], Keita Sato[3], Hirotaka Uzawa[4], Yasuo Seto[3], Yoshihiro Nishida[1*]

Address: [1]Department of Nanobiology, Graduate School of Advanced Integration Science, Chiba University, 1-33 Yayoi-cho, Inage-ku, Chiba 263−8522, Japan, [2]Department of Materials and Life Science, Shizuoka Institute of Science and Technology, 2200-2 Toyosawa, Fukuroi, Shizuoka 437-8555, Japan, [3]National Research Institute of Police Science, 6-3-1 Kashiwanoha, Kashiwa, Chiba 277-0882, Japan and [4]Nanosystem Research Institute, National Institute of Advanced Industrial Science and Technology (AIST), 1-1-1 Higashi, Tsukuba, 305-8565, Japan

Email: Hirofumi Dohi - hdohi@faculty.chiba-u.jp; Yoshihiro Nishida - YNishida@faculty.chiba-u.jp

*Corresponding author

Copies of ^{1}H and ^{13}C NMR spectra for compounds 2, 3 and 4.

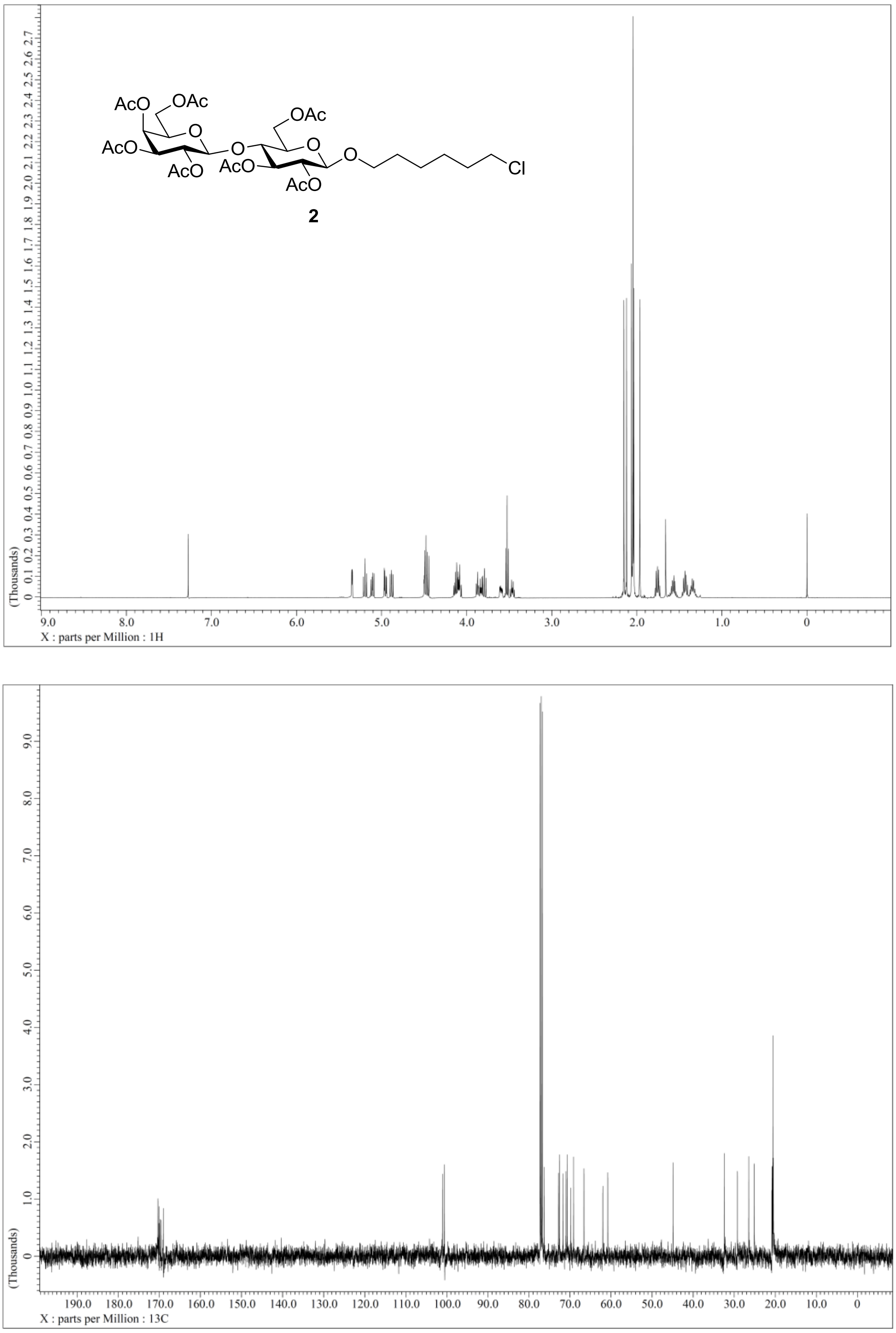

Figure S1. ^{1}H and ^{13}C NMR spectra of compound **2**.

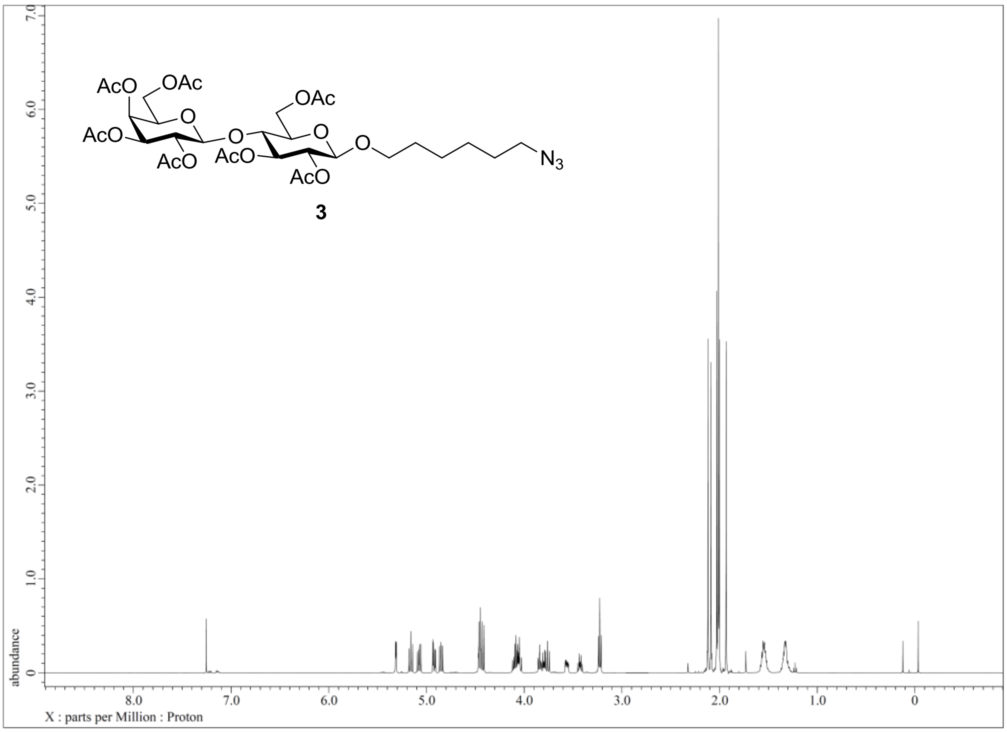

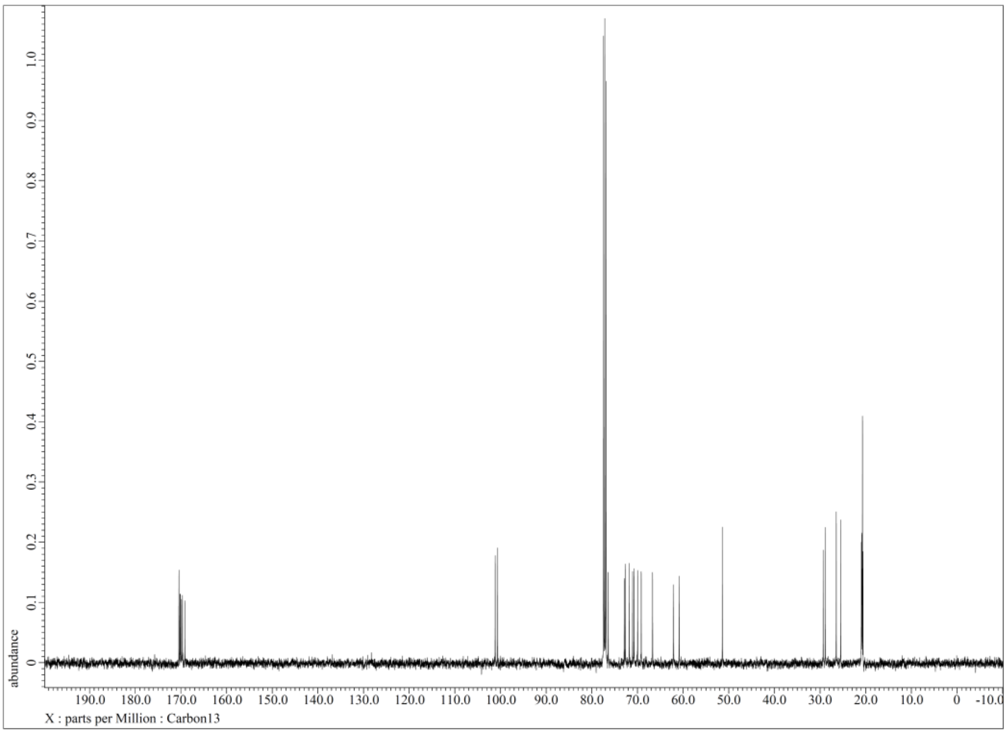

Figure S2. ^{1}H and ^{13}C NMR spectra of compound **3**.

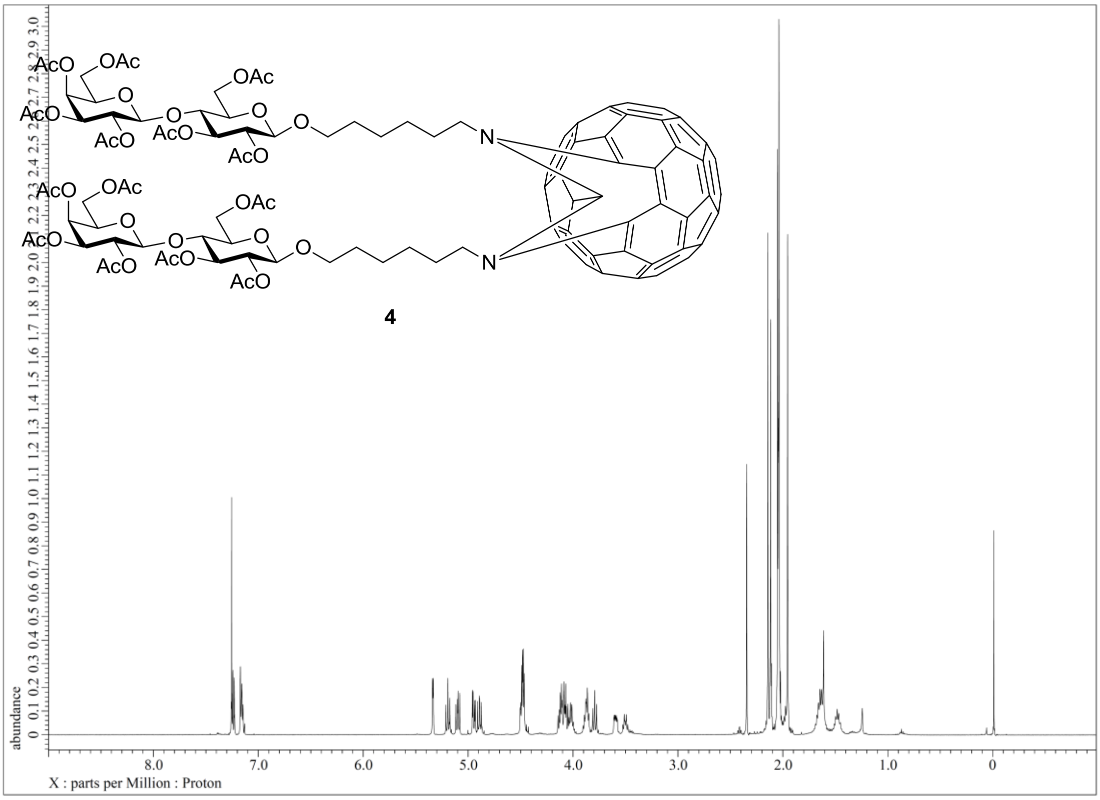

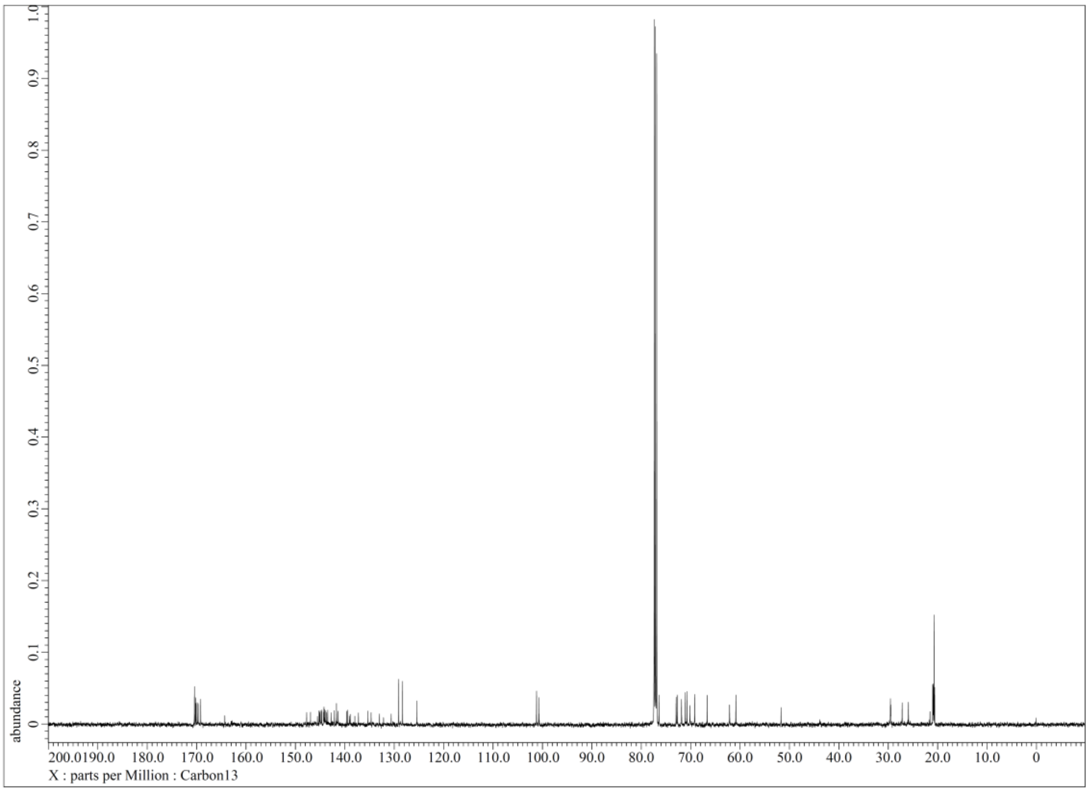

Figure S3. ^{1}H and ^{13}C NMR spectra of compound **4**.
